# A Virus Infecting *Hibiscus rosa-sinensis* Represents an Evolutionary Link Between Cileviruses and Higreviruses

**DOI:** 10.3389/fmicb.2021.660237

**Published:** 2021-05-03

**Authors:** Alejandro Olmedo-Velarde, John Hu, Michael J. Melzer

**Affiliations:** Department of Plant and Environmental Protection Sciences, University of Hawaii, Honolulu, HI, United States

**Keywords:** kitavirid, *Kitaviridae*, hibiscus, yellow blotch, HTS

## Abstract

Hibiscus (*Hibiscus* spp.) are popular ornamental and landscape plants in Hawaii which are susceptible to foliar diseases caused by viruses belonging to the genera *Cilevirus* and *Higrevirus* (family *Kitaviridae*). In this study, a virus infecting *H. rosa-sinensis* plants displaying foliar symptoms consistent with infection by a kitavirus, including yellow chlorotic blotches with a green perimeter, was characterized. The genome consisted of two RNAs 8.4 and 4.4 kb in length, and was organized most similarly to cileviruses, but with important distinctions. These included the location of the p29 homolog as the 3′-terminal open reading frame (ORF) of RNA2 instead of its typical locus at the 3′-end of RNA1; the absence of a p15 homolog on RNA2 and the adjacent intergenic region which also harbors small putative ORFs of unknown function; and the presence of an ORF encoding a 10 kDa protein at the 3′-terminal end of RNA1 that was also found to be present in the hibiscus green spot virus 2 genome. Spherical particles approximately 55–65 nm in diameter were observed in infected leaf tissue, and viral RNA was detected by reverse-transcription PCR in individual mites collected from symptomatic plants tentatively identified as *Brevipalpus yothersi*. Although phylogenetic analyses placed this virus between the higrevirus and cilevirus clades, we propose the tentative taxonomic placement of this virus, designated hibiscus yellow blotch virus (HYBV), within the genus *Cilevirus*.

## Introduction

The family *Kitaviridae* encompasses three genera of positive-sense ssRNA plant viruses: *Blunervirus*, *Cilevirus*, and *Higrevirus* (Melzer et al., 2018). Although related, there are considerable physical and genetic distinctions between members of the different genera. First, cile- and higreviruses are associated with a bacilliform virion, whereas a spherical virion has been observed for the lone blunervirus for which microscopy has been reported (Kitajima et al., 1974; Melzer et al., 2012; Hao et al., 2018). Second, cile-, higre-, and blunerviruses have bi-, tri-, and tetrapartite genomes, respectively (Quito-Avila et al., 2020). Third, different lineages of movement protein (MP) are present in the family: blunerviruses and cileviruses have a 3A/30K superfamily MP, whereas the lone higrevirus member possesses a triple gene block-like MP module (Quito-Avila et al., 2020). Finally, the replication-associated polyproteins are encoded by a single genomic RNA for cile- and higreviruses, but are split between two genomic RNAs for blunerviruses (Quito-Avila et al., 2013, 2020). As such, higreviruses and cileviruses share a closer phylogenetic relationship when conserved protein sequences are analyzed.

In recent years, several unclassified insect-infecting viruses, namely nelorpi- and sandewaviruses, Nunes et al. (2017) and arthropod viruses (Shi et al., 2016) have been characterized that resemble kitaviruses and appear to reside within the kitavirus clade. The RNA-dependent RNA polymerase (RdRp) of kitaviruses and that of the unsegmented negeviruses (nelorpi- and sandewaviruses) have a common phylogenetic origin and homologs of p24, a predicted virion membrane protein, are currently found only in these plant- and arthropod-infecting viruses (Kuchibhatla et al., 2014). The evidence of a common ancestry between these viruses has led to the hypothesis that plant-infecting kitaviruses arose from these arthropod-infecting viruses, with the arthropod vector being a potential origin (Nunes et al., 2017; Ramos-Gonzalez et al., 2020). The arthropod vector has only been confirmed for cileviruses, with *Brevipalpus* spp. (Acari: Tenuipalpidae) mites responsible for transmission in a persistent circulative, and likely propagative manner (Roy et al., 2015; Freitas-Astúa et al., 2018).

The family *Kitaviridae* is currently composed of five recognized species among the three genera: *Blueberry necrotic ring blotch virus* and *Tea plant necrotic ring blotch virus* (genus *Blunervirus*); *Citrus leprosis virus C* and *Citrus leprosis virus C2* (genus *Cilevirus*); and *Hibiscus green spot virus 2* (genus *Higrevirus*). The genomes of tomato fruit blotch virus, a putative blunervirus, and passion fruit green spot virus, a putative cilevirus, have also recently been described (Cuiffo et al., 2020; Ramos-Gonzalez et al., 2020). To better understand the diversity and evolutionary history of this family, it is imperative that additional members be described. In this study, we use high-throughput sequencing (HTS) to identify and characterize a new kita-like virus in Hawaii that infects *Hibiscus rosa-sinensis* in a non-systemic manner. This virus has distinctive genomic characteristics and phylogenetic analyses indicate it is an intermediate of cileviruses and higreviruses. Its bipartite genome suggests a tentative and temporary placement in the genus *Cilevirus*, and the name hibiscus yellow blotch virus (HYBV) is proposed.

## Materials and Methods

### Tissue Collection and Virus Indexing

**FIGURE 1:**
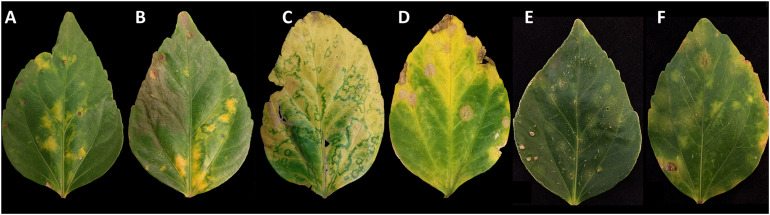
Variation in lesion symptoms displayed by *Hibiscus rosa-sinensis* leaves infected by hibiscus yellow blotch virus. Yellow blotches developed on mature leaves, and a dark green perimeter became visible as the leaves senesced. Lesions observed in panels **(A,B)** were predominant in July 2019; lesions observed in panels **(C,D)** were predominant in November 2019; lesions in panels **(E,F)** were predominant in March 2020. The lesions observed in panel **(D)** present a necrotic center that was sometimes observed in senescent leaves.

In July 2019, *Hibiscus rosa-sinensis* (L.) leaves displaying viral-like symptoms consistent with those caused by a brevipalpus-transmitted virus (BTV) (Kitajima et al., 2003) were collected from a single tree in Pearl City, Hawaii. Symptoms were characterized by yellow blotches surrounded by a green halo on both green and senescing leaves (Figure 1). Additional samples from the same tree were collected in in November 2019 and March 2020. Leaf samples displaying green ringspot symptoms consistent with BTV infection in Hawaii (Melzer et al., 2013) were subsequently collected from six *Hibiscus* spp. trees growing within 1.6 km of the original *H. rosa-sinensis* tree.

To determine if the *H. rosa-sinensis* tree was infected with hibiscus green spot virus 2 (HGSV-2) and the hibiscus strain of citrus leprosis virus C2 (CiLV-C2H), which have been associated with similar, yet distinct, symptoms in Hawaii’s hibiscus plants, virus-specific RT-PCR assays were performed using existing protocols (Melzer et al., 2012, 2013).

### Genome Sequencing

Double stranded RNAs (dsRNAs) were extracted from ∼5 g of symptomatic leaf tissue collected from the *H. rosa-sinensis* tree in Pearl City using either CF-11 (Whatman, Maidstone, United Kingdom) or C6288 (Sigma, St. Louis, MO, United States) cellulose chromatography (Morris and Dodds, 1979) and resolved by 1X TBE-1% agarose gel electrophoresis. Using dsRNAs as template, randomly amplified cDNAs were generated (Melzer et al., 2010) and prepared for HTS using a Nextera XT DNA Library Prep kit (Illumina, San Diego, CA, United States). HTS was performed on an Illumina MiSeq 2 × 300 bp (V2) platform at the University of Hawaii Advanced Studies in Genomics, Proteomics, and Bioinformatics (ASGPB) Laboratory. Genome assembly and bioinformatic analyses were performed as described (Olmedo-Velarde et al., 2019). Briefly, paired-end reads were trimmed, and quality filtered using Trimmomatic 0.35.3 (Bolger et al., 2014). Trinity 2.2.0 (Grabherr et al., 2011) produced *de novo* assembled contigs that were annotated using BLASTX search (Altschul et al., 1997) against the viral genome database^1^. Contiguous sequences (contigs) with similarity to cilevirus sequences were then used as reference for an iterative mapping approach (Dey et al., 2019) using Geneious mapper plug-in implemented in Geneious v. 10.1.3 (Kearse et al., 2012) and raw reads. A group of overlapping primer sets (Supplementary Table 1) that were designed as detailed below and based on the contigs of HYBV were used to validate the HTS output and bridge gaps by RT-PCR. Termini were characterized by 5′ RACE as detailed by Navarro et al. (2018) on poly-A tailed dsRNAs and poly-dG-tailed cDNAs that were generated using *E. coli* Poly(A) Polymerase (New England Biolabs, Ipswich, MA, United States) and Terminal Deoxynucleotidyl Transferase (Thermo Fisher Scientific, Waltham, MA, United States), respectively. 3′ RACE was performed by RT-PCR using an oligo-dT primer to target the poly-A tracts at the 3′-end of both RNA 1 and 2. Primers employed for genome validation and RACE experiments are detailed in Supplementary Table 1. Amplicons were cloned into pGEM-T Easy (Promega, Madison, WI, United States) and three to five clones were sequenced.

### Genomic and Proteomic Analyses

The NCBI ORFfinder program^2^ was used to identify putative open reading frames (ORFs) *in silico*. Conserved domains were predicted using either the NCBI conserved domain search tool^3^ or HMMSCAN^4^ implemented in HMMER (Potter et al., 2018). HMMSCAN was also used for the prediction of transmembrane helices, signal peptides, coiled coils and protein disorders. Protein transmembrane helices were also predicted and visualized using TMHMM (Krogh et al., 2001) implemented in Geneious 10.1.3. Signal peptides for protein cleavage were predicted using SignalP-5.0^5^.

BLASTP searches were used to retrieve protein homologs and infer putative function. Furthermore, putative orphan proteins showing no homology to any protein in any database were aligned using their structural information and the Expresso algorithm (Armougom et al., 2006) implemented in T-Coffee^6^ (Di Tommaso et al., 2011). Protein sequence alignment was evaluated using TCS (Chang et al., 2014) implemented in T-Coffee.

Pairwise protein sequence comparisons using orthologous sequences retrieved from GenBank were performed using LALIGN^7^ (Huang and Miller, 1991). In addition, the percentage pairwise protein identities of multiple alignments of the replication-associated polyproteins, putative MPs, and the putative structural p23 orthologs were determined using sequence demarcation tool (SDT) 1.2 (Muhire et al., 2014) and the MUSCLE algorithm implemented in SDT 1.2.

The 5′ and 3′ untranslated regions (UTR) of all isolates of recognized and putative/newly described kitaviruses, including HYBV, were analyzed. Intra-species UTR sequences underwent multiple alignment using the ClustalW algorithm (Thompson et al., 1994) implemented in Geneious 10.1.3 (Kearse et al., 2012). Nucleotide composition such as A/T% and their length were determined for all the 5′ and 3′ UTR. Conserved 5′ and 3′ termini were identified, and their length, nucleotide identity as well as the best consensus sequence were manually determined based on these alignments.

### Phylogenetic Analyses

Phylogenetic relationships between HYBV and members of the family *Kitaviridae*, *Virgaviridae*, *Bromoviridae*, *Closteroviridae*, and negeviruses were inferred using the amino acid sequences of multiple proteins/domains conserved among some or all of these viruses. Multiple protein alignment was performed with ClustalW (Thompson et al., 1994) implemented in MEGA 7.0.25 (Kumar et al., 2016). Ambiguous positions for each alignment were curated using Gblocks 0.91b^8^ (Talavera and Castresana, 2007). The best model of protein evolution for each alignment was used to generate a maximum likelihood tree with 1,000 bootstrap repetitions. Bayesian phylogeny was inferred using BEAST 2.6.2 (Bouckaert et al., 2019) and the best model of protein evolution with three Markov chain Monte Carlo runs of 10,000,000 generations with sampling every 1,000 trees. The runs were combined using LogCombiner in BEAST and 10% of the sample trees were discarded as “burn-in.” Tracer 1.7.1 (Rambaut et al., 2018) was used to confirm sample sizes were above 200 for all the parameters. Maximum clade credibility and posterior probabilities were annotated using TreeAnnotator in BEAST 2.6.2. The output trees were visualized in FigTree 1.4.4.

### Transmission Electron Microscopy

Using leaf samples collected in March 2020 from the Pearl City *H. rosa-sinensis* tree, ultra-thin sections for transmission electron microscopy were prepared and observed as described (Melzer et al., 2012; Olmedo-Velarde et al., 2019). Briefly, 1 × 2 mm pieces excised from asymptomatic tissue and the margin of lesions from symptomatic leaves were fixed using 2% glutaraldehyde and 2% paraformaldehyde, and post-fixed using a 0.1 M sodium cacodylate solution containing 1% osmium tetroxide. Ultra-thin sections were embedded, stained with uranyl acetate and lead citrate.

Partially purified virion preparations were obtained as described by Colariccio et al. (2000). Briefly, ∼5 g of symptomatic *H. rosa-sinensis* leaf tissue was powdered with liquid nitrogen and mixed with 20 mL of extraction buffer (0.05 M phosphate buffer pH 7.0 containing sodium DIECA, 0.1% (w/v) ascorbic acid and 0.02 M sodium sulphite) for 30 min. After clarification, 0.5% (w/v) sodium chloride and 6% (w/v) 6000 polyethylene glycol were added to the suspension, and partially purified virions were pelleted by centrifugation at 8,000 × *g* for 10 min. The pellet was resuspended with 1 mL of extraction buffer overnight. All steps were performed at ∼4°C. A 10-fold dilution of the partially purified virion preparations was negatively stained on formvar/carbon-coated grids using 1% uranyl acetate (UA) or 1% phosphotungstic acid (PTA). A density plot based on the diameter of observed particles was created with ggplot2 (Wickham, 2016).

Ultra-thin sections and negatively stained partially purified virion preparations were viewed with a HT7700 120 kV transmission electron microscope (Hitachi High Technologies America Inc., Dallas, TX, United States) at the University of Hawaii Biological Electron Microscope Facility.

### Virus Detection

Total RNA was extracted from 100 to 200 mg of plant samples collected in July 2019 using NucleoSpin RNA Plus kit (Macherey-Nagel, Düren, Germany) or Spectrum Plant Total RNA kit (Sigma-Aldrich, St. Louis, MO, United States). These RNA extracts were reverse transcribed into cDNA using random primers and M-MLV reverse transcriptase (Promega) using the manufacturer’s protocol. Two microliters of cDNAs were tested by endpoint PCR using GoTaq Green Master Mix (Promega). Virus-specific primer sets for HYBV were designed to target the RNA-dependent RNA polymerase (RdRp) domain and p10 in RNA 1, and p33 in RNA 2, respectively. Primer3 (Untergasser et al., 2012) was used for the primer design with consideration of thermodynamic primer features (Arif and Ochoa-Corona, 2013). HYBV-RdRp-F/R, HYBV-p10-F/R, and HYBV-p33-F/R (Supplementary Table 1) were used for specific detection of HYBV in endpoint RT-PCR assays using 0.5 μM as final primer concentration and 55°C as the annealing temperature. Furthermore, one-step quantitative reverse transcription (RT-qPCR) assays were implemented using HYBV-RdRp-F/R, and CiLV-C2-RdRP-F/R and HGSV-2-RdRp-F/R (Supplementary Table 1). The two latter primer sets were designed as described above and based on a consensus sequence of an alignment of the RdRp sequences of CiLV-C2 and HGSV-2 available in GenBank as well as sequences from additional isolates of CiLV-C2 and HGSV-2. One-step RT-qPCR assays were implemented using iScript One-Step RT-PCR Kit with SYBR Green (Bio-Rad, Hercules, CA, United States) and 0.125 μM of each primer. Each reaction was performed in three replicates. Cycling parameters for all RT-qPCR assays consisted of cDNA synthesis at 50°C for 40 min. Later, an initial denaturation was performed at 95°C for 1 min, followed by 35 cycles of denaturation at 95°C for 10 s and an annealing-extension step at 60°C for 1 min during which time data was collected. Melt curve analysis was performed as follows: 95°C for 1 min, pre-melting conditioning at 60°C for 1 min followed by a melting temperature cycle range from 60°C to 95°C. Positive cDNA controls, specific for each virus, and non-template controls (DEPC-treated water) were used in all of the assays.

### Mite Barcoding and Virus Detection in Flat Mites

Four individual flat mites (*Brevipalpus* spp.), collected in 2020 from the symptomatic *H. rosa-sinensis* tree in Pearl City were used for direct reverse transcriptase (DRT)-PCR assays (Druciarek et al., 2019). Briefly, individual *Brevipalpus* mites were introduced into a PCR tube containing 10 μl of water and random hexamer primers and crushed using a needle under a dissecting microscope. Then, cDNA was synthesized using random primers and SuperScript III reverse transcription kit (Thermo Fisher Scientific) using the manufacturer’s instructions. Two microliters of ten-fold diluted cDNA reactions were used in endpoint PCR for DNA barcoding and internal PCR control using the 28S rRNA primers, D1D2w2: 5′-ACAAGTACCDTRAGGGAAAGTTG-3′, 28Sr0990: 5′-CCTTGGTCCGTGTTTCAAGAC-3′ (Sonnenberg et al., 2007; Mironov et al., 2012; Druciarek et al., 2019) that produce a ∼700 bp expected amplicon. The cytochrome oxidase unit I (COI) gene was additionally amplified using the COI primers, DNF: 5′-TACAGCTCCTATAGATAAAAC-3′, DNR: 5′-TGATTTTTTGGTCACCCAGAAG-3′ (Navajas et al., 1996) that produce a ∼450 bp expected amplicon. All DNA barcoding PCR assays were performed using Q5 High Fidelity DNA Polymerase (New England Biolabs, Ipswich, MA, United States). Furthermore, endpoint RT-PCR assays using primers HYBV-RdRp-F/R, HYBV-p10-F/R and HYBV-p33-F/R were performed as detailed above to detect the presence of HYBV in the mite specimens. All amplicons were gel extracted, purified and bi-directionally sequenced or cloned into pGEM-T Easy with three clones sequenced per amplicon.

## Results

### Symptom Monitoring and Virus Indexing

The expression of symptoms in a *H. rosa-sinensis* plant consistent with BTV infection was periodically observed from July 2019 to March 2020. The predominant symptomology varied over this 9-month period, ranging from faint, circular chlorotic blotches (March 2020), chlorotic blotches with a green perimeter (July 2019), to circular necrotic lesions (November 2019) that may represent an advanced stage of the faint, circular chlorotic blotches (Figure 1). Symptoms were often most dramatic in senescing leaves. Total RNA extracted from symptomatic leaf tissue (Figure 1) tested negative for CiLV-C2 and HGSV-2 in two-step RT-PCR assays. Positive and non-template (water) controls performed as expected (data not shown). Subsequent testing using the two-step RT-PCR assay and primers HYBV-RdRp-F/R revealed that all symptomatic tissues were positive for HYBV (data not shown).

### Molecular Characterization of Hibiscus Yellow Blotch Virus

Agarose gel electrophoresis revealed the presence of two dsRNA bands of ∼8 and ∼4.5 Kbp isolated from symptomatic *H. rosa-sinensis tissue* (Supplementary Figure 1). HTS of a library generated from these dsRNAs produced ∼24 M paired-end reads that were *de novo* assembled into 2,178 contiguous sequences (contigs). Of these, 27 showed similarity to cileviruses and higreviruses. Iterative mapping of raw reads and reassembling of these contigs led to the generation of three larger contigs of 8,127, 3,676, and 507 bp in length. BLASTX searches showed the three contigs putatively coded proteins showing low to moderate identity to the replication-associated polyprotein coded by RNA 1 of cileviruses and HGSV-2, p61 through p24 coded by RNA 2 of cileviruses, and to p29 coded by RNA 1 of cileviruses, respectively. Using the sequence of the 8,127 bp contig, 5′ and 3′ RACE was performed to complete the RNA 1. Using the sequence of the 3,676 bp contig, 5′ RACE was used to determine the 5′ terminal sequence, and 3′ RACE using primer 909 (Supplementary Table 1) resulted in a ∼1,620 bp amplicon which included the sequence of the 507 bp contig. RT-PCR using primer 909 and HYBV-p33-R (Supplementary Table 1) validated the 3′ RACE result by bridging the ∼307 nt sequence gap between the 3,676 and 507 bp contigs.

**FIGURE 2:**
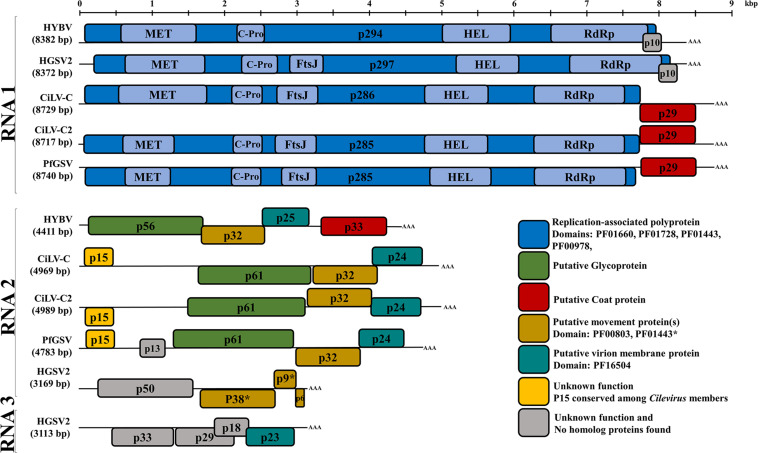
Genome organization comparison between hibiscus yellow blotch virus (HYBV) and *Cilevirus* and *Higrevirus* members. Boxes represent open reading frames (ORFs), and similarly colored ORFs encode proteins of similar function as indicated. RNA 1 (8382 bp) contains two ORFs coding for the replication-associated polyprotein (p294) and a protein with unknown function (p10). RNA 2 (4411 bp) contains four ORFs coding for the putative glycoprotein (p56), putative movement protein (p32), putative virion membrane protein (p25) and putative coat protein (p33). Cileviruses represented: citrus leprosis virus C (CiLV-C) and CiLV-C2, and the proposed cilevirus, passion fruit green spot virus (PfGSV). Higreviruses represented: hibiscus green spot virus 2 (HGSV2). MET, methyltransferase; C-Pro, cysteine like protease; FtsJ, methyltransferase; HEL, helicase; RdRp, RNA-dependent RNA polymerase; AAA, poly(A) tail. Scale at top of figure indicates approximate size in kilobase pairs.

Excluding the poly-A tails at their 3′ end, RNA 1 and RNA 2 of HYBV were 8,382 and 4,411 nt, respectively (Figure 2). The 8,382 bp RNA 1 molecule (GenBank accession MT472637) had a large ORF that putatively encoded a 294 kDa replication-associated polyprotein of 2,645 amino acids (aa). This putative polyprotein possessed viral methyltransferase (MET; PF01660, aa residues 144–495), cysteine protease (C-Pro; PF02338, aa residues 681–816), viral helicase 1 (HEL; PF01443, aa residues 1,640–1,932) and RdRp 2 (PF00978, aa residues 2,148–2,587) domains (Figure 2). This polyprotein was most similar to that of CiLV-C2H, with an identity of 37% (Table 1 and Supplementary Figure 2). The individual MET, C-Pro, HEL and RdRp 2 domains were 49%, 39%, 44% and 61% identical to those of CiLV-C2H, respectively. No transmembrane helices were found in this protein, however, coiled coil and disorder regions were identified at aa residues 1620–1640 and 1461–1468, respectively. In the 3′-terminal region of RNA 1, an 85 aa ORF was identified that putatively encoded a 10 kDa protein harboring two transmembrane helices. This protein of unknown function, designated p10, showed no similarity to any protein in the current databases as determined by a BLASTP search. However, it did resemble the size, genomic location and secondary structure of a previously undescribed ORF of HGSV-2 (Figure 2 and Supplementary Figure 3A). A structural alignment of the two putative proteins obtained using Expresso implemented in T-Coffee revealed an identity of 20% (Table 1 and Supplementary Figures 3B,C).

**TABLE 1:** Percent amino acid identities between orthologous proteins of hibiscus yellow blotch virus (HYBV) and *Kitaviridae* members: higreviruses include: hibiscus green spot virus 2 (HGSV-2); cileviruses include: citrus leprosis virus C (CiLV-C) and CiLV-C2 (with isolates indicated), and the proposed cilevirus, passion fruit green spot virus (PfGSV); blunerviruses include, blueberry necrotic ring blotch virus (BNRBV) and tea plant necrotic ring blotch virus (TPNRBV), and the putative blunervirus tomato fruit blotch virus (TFBV).

Protein	HGSV2^2^	CiLV-C (CRD)	CiLV-C (SJP)	CiLV-C2 (Citrus-Co)	CiLV-C2 (Hibiscus-HI)	PfGSV (Snp1)	BNRBV	TPNRBV	TFBV
**RNA1-Polyprotein**	33.9	35.9	36.1	36.3	**36.6**	36.4	32.1	26.3	26.0
**RNA1-p10**	19.7	–	–	–	–	–	–	–	–
**RNA2-p56**^1^	–	20.8	**22.3**	20.7	19.5	19.1	–	–	–
**RNA2-p32**	– ^2^	40.6	39.6	**45.9**	44.4	45.3	36.6	32.7	34.1
**RNA2-p25^1^**	27.7	**40.0**	39.4	38.2	37.3	39.8	23.2	26.9	22.4
**RNA2-p33^1^**	–	20.3	23.1	25.6	**32.8**	28.1	–	–	–

*Orthologous proteins showing the highest identity to the HYBV proteins are bold. ^1^p56, p25, and p33 of HYBV shows resemblance to p61, p24 and p29 of cileviruses, respectively. ^2^HGSV-2 codes for a triple gene block-like movement proteins rather than 3A or 30K movement proteins (Melzer et al., 2018).*

The 4,411 bp RNA 2 molecule (MT472638) possessed four ORFs. From the 5′ end, the first ORF putatively encoded for a 485 aa protein with a molecular weight of 56 kDa. This protein possessed three transmembrane helices in the C-terminus and shared low homology (19–22% aa identity) to the cilevirus p61 (Table 1), which represents the putative glycoprotein. A signal peptide that has been predicted for the p61 of some cileviruses (Ramos-Gonzalez et al., 2020) was also identified in the HYBV homolog, with a predicted cleavage site at Arg_20_/Val_21_. The second ORF putatively encoded for a 301 aa protein (32 kDa) in which several disorder regions were identified with the largest located in the C-terminus, at aa residues 270–301. This protein has a conserved domain (PF00803, aa residues 6–227) of viral movement proteins (MP) and likely represents the MP of HYBV. It shared moderate (33–46%) identity with orthologs of kitaviruses (Table 1). The third ORF putatively encoded a 230 aa protein, p25, and harbors an SP24 conserved domain (PF16504, aa residues 33–156) which possesses four transmembrane helices. SP24 is present in kitavirids, negeviruses and chroparaviruses, with the latter two being taxons of insect-specific viruses (Kuchibhatla et al., 2014). Two disorder regions were found in the C-terminus of the HYBV p25, and the protein shared moderate (23–40%) identity with orthologs of recognized kitaviruses (Table 1). The 3′-terminal ORF putatively encoded a 312 aa protein orthologous (20–33% aa identity) to p29 encoded on RNA1 of cileviruses (Table 1), which is a predicted coat protein (Leastro et al., 2018). A long disorder region was identified in aa 52–163 of the p33.

### Transmission Electron Microscopy

**FIGURE 3:**
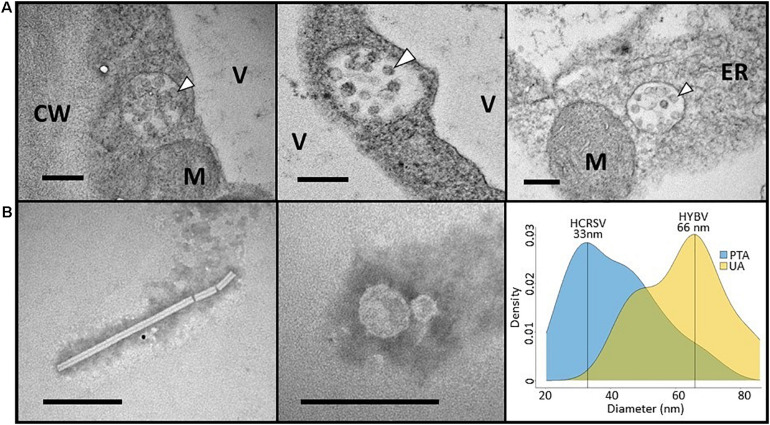
**(A)** Electron micrographs of hibiscus yellow blotch virus-infected *Hibiscus rosa-sinensis* leaves containing aggregates of electron dense spherical structures (arrowheads) typically between 50 and 60 nm in diameter. Similar structures were not observed in healthy tissue. CW, cell wall; ER, endoplasmic reticulum; M, mitochondrion; V, vacuole. **(B)** Electron micrographs of a rod-shaped virion (left) and spherical particles (center) partially purified from *H. rosa-sinensis* leaves co-infected with hibiscus latent Fort Pierce virus (HLFPV), hibiscus chlorotic ringspot virus (HCRSV), and hibiscus yellow blotch virus (HYBV). The rod-shaped virion is consistent with the HLFPV virion, whereas the spherical particles could represent HCRSV and HYBV virions. A density plot was generated to determine the frequency of spherical particles stained with either phosphotungstic acid (PTA) or uranyl acetate (UA) based on their diameter (right). Particle diameters of 33 and 66 nm were most frequently recorded, which may correspond to HCRSV and HYBV virions, respectively. Bar = 200 nm for all micrographs.

Ultra-thin sections prepared from foliar lesions revealed the presence of small congregations of spherical structures approximately 50–60 nm in diameter (Figure 3). These spherical structures, contained in cytosolic vesicles, were typically in close proximity to the endoplasmic reticulum. Although not common in cells from symptomatic tissue, these structures were not observed in ultra-thin sections obtained from asymptomatic leaves. Despite extensive examination of six thin sections from both symptomatic and asymptomatic leaves, neither electron-dense viroplasm or bacilliform virus-like particles were observed.

Abundant rod-shaped and spherical particles were observed in the partially purified, negatively stained virus preparations. Rod-shaped virions were approximately 20 nm in width and up to 370 nm in length, and were consistent with tobamovirus virion morphology (Figure 3). Spherical particles encompassing a wide range of diameters were observed. A density plot based on the measurement of 156 particles indicated diameters of 33 and 66 nm were most prevalent, and these particle sizes were most readily observed with PTA and UA staining, respectively (Figure 3).

### Phylogenetic Placement of Hibiscus Yellow Blotch Virus

**FIGURE 4:**
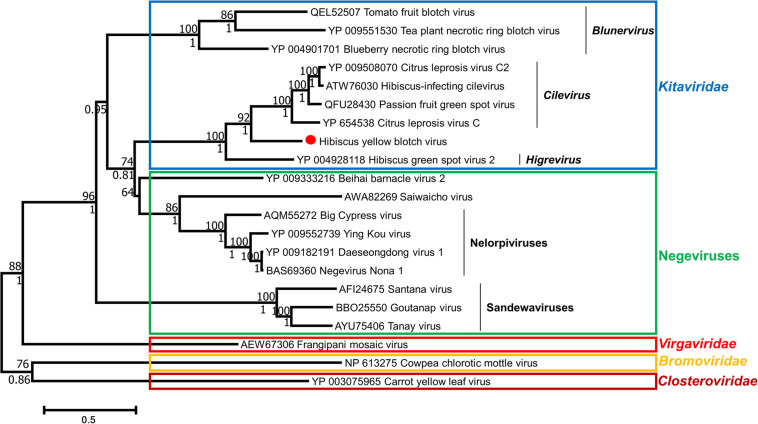
Phylogenetic relationships among members of the families *Kitaviridae*, *Virgaviridae*, *Bromoviridae*, *Closteroviridae* and negeviruses based on an RNA-dependent RNA polymerase (RdRp) multiple protein alignment using CLUSTAL and inferred using maximum likelihood algorithm implemented in MEGA 7.0.25. Bootstrap values generated by maximum likelihood are shown above the branches after 1000 repetitions. Posterior probabilities that were calculated using Bayesian inference with three Markov Chain Monte Carlo runs of 10,000,000 generations and implemented in BEAST 2 are shown under the branches. Missing values indicate values below 50 (bootstrap) or 0.7 (posterior probability). GenBank accession numbers are provided prior to each virus name. Scale at bottom indicates the number of substitutions per given branch length.

Phylogenies of HYBV were inferred with two character-based algorithms: Maximum likelihood and Bayesian inference. Both algorithms predicted a similar relationship between HYBV and other viruses for each protein sequence analyzed. For the RdRp and p24 proteins, HYBV formed a monotypic lineage between the cilevirus and higrevirus clades (Figure 4 and Supplementary Figure 4). For the p61 and p29 proteins, which only have homologs in cileviruses, HYBV consistently formed a basal branch (Supplementary Figure 4). For the MP, which has homologs in cileviruses, blunerviruses, and other plant-infecting viruses, HYBV was placed between the cilevirus and blunervirus clades (Supplementary Figure 4). Furthermore, in the RdRp and p24 phylogenies, *Kitaviridae* members and negeviruses formed a monophyletic group sharing a common ancestor (Figure 4 and Supplementary Figure 4).

### Protein Comparisons Between Genera in the *Kitaviridae*

To evaluate genetic divergence between HYBV and cileviruses in the context of inter-genera divergence within the family *Kitaviridae*, protein sequence identity matrices of the replication-associated polyprotein, p24, and putative MP of kitavirids were obtained using SDT 1.2. The matrices show overall low to moderate protein identity levels, from 21 to 45%, for the three proteins of HYBV with their kitavirid homologs (Table 1 and Supplementary Figure 2). Cileviruses, including the recently characterized passion fruit green spot virus (PfGSV), present high protein intra-genus identities ranging from 59 to 81, 62 to 89, and 51 to 75% for the replication-associated polyprotein, p24 and MP, respectively. However, cileviruses present a low protein identity, not surpassing 45%, with their homologs of HYBV, HGSV-2 and blunerviruses. The sole higrevirus, HGSV-2, has low protein identity ranges of 24–36 and 22–33% with other kitavirids for the replication-associated polyprotein and p24, respectively. Blunerviruses also show low protein identity ranges of 24–36, 20–31, and 29–38% with other kitavirids, including other blunerviruses, for the replication-associated polyprotein, p24 and MP, respectively. In general, kitavirids present broad protein identity ranges of 24–81%, 20–89%, and 29–75% for the replication-associated polyprotein, p24 and MP.

### Analyses of Untranslated Regions of Kitavirids

Multiple nucleotide sequence alignments revealed that cileviruses, HGSV-2, and HYBV share common features in their UTR, including AT richness. They possess conserved 5′ and 3′ termini, and conserved long 3′ UTRs in all of their genomic segments (Supplementary Table 2). All their 5′ termini start with a G or C, while most of their 3′ termini end with a C. Most of their UTR length are uniform among intra-species genomic segments. Furthermore, cileviruses present a long conserved 3′ termini of ∼122 nucleotides and the last three conserved nucleotides are GAC. Similar to cileviruses, HYBV possesses a long conserved 3′ terminus of 171 nucleotides and the last three conserved nucleotides for each RNA segment are GCC. HGSV-2 possesses a shorter conserved 3′ terminus of 78 nucleotides with the last three not clearly conserved. Whereas, blunerviruses also presented AT-rich 5′ and 3′ UTRs, but shorter semi-conserved termini in their genomic segments. Their UTR lengths are variable among intra-species genomic segments.

### Hibiscus Yellow Blotch Virus Detection in Flat Mites

Four individual flat mites (*Brevipalpus* spp.) were collected from a symptomatic *H. rosa-sinensis* infected with HYBV. HYBV RNA 1 and RNA 2 were amplified by DRT-PCR in two of these four mites using three different HYBV-specific primer sets (Supplementary Figure 5). Direct sequencing showed the three sequences share 100% nucleotide identity to the RdRp and p10 regions in RNA 1 and p33 in RNA 2 of HYBV isolate present in the from the symptomatic *H. rosa-sinensis* tree. Using 28s rRNA primers, a prominent ∼700 bp amplicon of expected size was produced from each of the four individual mites using DRT-PCR, as well as a faint ∼340 bp amplicon which was sequenced and found to be non-specific (Supplementary Figure 5). Direct sequencing of the ∼700 bp 28S rRNA amplicon for those two individual mites, and a pairwise alignment showed that both sequences shared 100% nucleotide identity. A BLASTN search using the consensus sequence (MT812697) showed that it shares 99.7% nucleotide identity to *B. yothersi* (MK293649) with 88% query coverage. Direct sequencing of the 28S rRNA region was performed for the other two mites in which HYBV was not detected. A pairwise alignment revealed that both sequences shared 100% nucleotide identity. A BLASTN search of a consensus of both sequences (MT812698) showed that it shares 98.4% and 98.1% nucleotide identity to *B. azores* (MK919272) and *B. feresi* (MK919273), respectively, both with 89% query coverage. Furthermore, direct sequencing of the ∼450 bp amplicons generated using the COI primers (data not shown) further corroborated the identity of *B. yothersi* after a BLASTN search that show the consensus sequence (MT796740) shares 99.3% nucleotide identity to *B. yothersi* (KP180426) with 100% query coverage. A consensus sequence (MT796741) for the COI gene of the two other mites, in which HYBV was not detected, showed 100% identity to *B. obovatus* (DQ450495) after a BLASTN search with a 90% query coverage.

### Natural Mixed Infections in Hibiscus

In addition to HYBV, HTS data from the dsRNA library also identified the complete genomic sequences of hibiscus chlorotic ringspot virus (HCRSV, *Betacarmovirus*) and hibiscus latent Fort Pierce virus (HLFPV, *Tobamovirus*) in the Pearl City sample using *de novo* assembly and an iterative mapping approach. The genome of HCRSV from the sample was 3,969 nt in length (MT512573) and assembled from 1,386,006 reads with and average depth of 33,785. The genome shared 95.4% nucleotide identity to an HCRSV isolate from Singapore (X86448). No dsRNAs associated with this virus were observed following agarose gel electrophoresis, however, any dsRNAs of this size would have been obscured by the loading dye (Supplementary Figure 1). The RdRp, p2, p3, and CP of HCRSV shared 95%, 95.7% and 92.3% protein identity, respectively, to those of the HCRSV isolate from Singapore, while the CP shared 99.4% protein identity to that of HCRSV isolate SB01 from Brazil (AZL87708). The genome of HLFPV was 6,408 nt in length (MT512572) assembled from 1,667,706 reads with an average depth of 35,941. The genome shared 99.4% nucleotide identity to HLFPV isolate J from Japan (AB917427). This genome size was consistent in size with a faint dsRNA observed following agarose gel electrophoresis (Supplementary Figure 1) Replication-associated polyprotein, MP and CP of HLFPV show 98.5%, 99.7%, and 97.5% protein identity, respectively, to those of HLFPV isolate J (AB917427).

Samples from six *Hibiscus* spp. plants designated A-F displaying symptoms typical of BTV infection (Kitajima et al., 2003) were collected within 1.6 km of the original HYBV-infected tree (Supplementary Figure 6). The presence of CiLV-C2, HGSV-2 and HYBV, was assessed by one-step RT-qPCR and two-step RT-PCR assays. Three out of six of the samples (A, B, and D) tested positive for HYBV in both PCR assays. Only one sample (A), which also tested positive for HYBV, tested positive for CiLV-C2 in both PCR assays. None of the samples tested positive for HGSV-2. In all of the assays, positive and non-template controls tested as expected (Supplementary Figure 7). The genetic diversity of HYBV in these samples was assessed for RNA1 and RNA2 by sequencing amplicons generated using primer sets HYBV-RdRp-F/HYBV-p10-R and 909/HYBV-p33-R, respectively (Supplementary Table 1). These amplicons were 100% identical for RNA1 and >99.6% identical for RNA2.

## Discussion

The *Kitaviridae* family is a recently created taxon currently comprised of three genera of plant pathogenic viruses: *Blunervirus*, *Cilevirus*, and *Higrevirus* (Melzer et al., 2018; Quito-Avila et al., 2020). Kitavirids share an evolutionary history based on the three proteins they commonly possess: replication-associated polyprotein, p24 protein, and in the case of blunerviruses and cileviruses, the MP. Additionally, mites (eriophyid and flat or false spider) have been reported as the putative or confirmed vectors of these viruses (Burkle et al., 2012; Rodrigues et al., 2016). Interestingly, systemic infection of their plant host is rare; most kitavirid infections of plants are restricted to localized lesions (Quito-Avila et al., 2020). Several *Kitaviridae* members have been characterized through the use of HTS (Quito-Avila et al., 2013; Roy et al., 2013; Hao et al., 2018; Ramos-Gonzalez et al., 2020) which is an increasingly important and efficient technique for virus discovery and characterization, including in numerous studies in different agricultural systems (Villamor et al., 2019).

In this study, we have characterized a new kitavirid infecting *H. rosa-sinensis* in Hawaii using HTS of a dsRNA library. The symptoms displayed on leaves infected with this virus resemble those associated with infection by cileviruses and other kitavirids (Figure 1) (Melzer et al., 2013; Rodrigues et al., 2016). RT-PCR assays specific for CiLV-C2 and HGSV-2 indicated these two kitavirids, which commonly infect hibiscus in Hawaii, were absent, suggesting the presence of another pathogen. HTS and bioinformatic analyses allowed the complete bipartite genome of this new kitavirid to be characterized. The genomic RNAs, determined to be 8.3 and 4.4 kb in size, were consistent with the dsRNAs observed by agarose gel electrophoresis (Supplementary Figure 1). RNA 1 codes for a RdRp and a putative p10 protein with unknown function. RNA 2 codes for p56, p32, p25 and p33 proteins that likely represent the putative glycoprotein, putative movement protein, putative virion membrane protein and putative coat protein, respectively. This genomic organization resembles that of cileviruses, but with some key differences (Figure 2). First, p33 is located in the 3′ region of RNA 2, but the homolog of this protein in cileviruses (p29) is located in the 3′ region of RNA 1. These represent the putative viral coat proteins (Leastro et al., 2018), and this observation suggests a gene rearrangement event in an ancestor of these viruses. Second, p15 is the most variable conserved orphan protein present in the 5′ region of RNA 2 of the three classified and putative *Cilevirus* species (Ramos-Gonzalez et al., 2020). This new kitavirid lacks a p15 homolog in the 5′ region of its RNA 2. The absence of a p15 homolog was supported by an inability to identify this putative protein *in silico*, using 5′ RACE, and through degenerate RT-PCR assays targeting conserved amino acid sequences identified following the alignment of cilevirus p15 sequences. Based on sequence comparisons and recombination analyses of the 5′ end of the cilevirus RNA 2, it has been previously suggested that the high nucleotide variability present in that region is the result of continuous illegitimate (non-homologous) recombination processes that may occur at the inter-species level (Ramos-Gonzalez et al., 2016). Therefore, recombination processes may have contributed to the loss of a p15 homolog in an ancestor of HYBV. Finally, a 3′-terminal ORF encoding a putative 10 kDa protein is present in HYBV RNA 1. Although the predicted p10 protein shares no homology to viral proteins in in GenBank, a pairwise protein structural alignment of the HYBV p10 and the product of a similarly sized and positioned ORF encoded by HGSV-2 using EXPRESSO T-Coffee revealed they share statistically significant structural similarities. A global pairwise alignment showed they share 19.7 % protein identity. Furthermore, both p10 proteins of HYBV and HGSV-2 possess two transmembrane domains (Supplementary Figure 3). This suggests these putative orphan proteins of HYBV and HGSV-2 are either distant orthologs or represent structural convergence of two unrelated proteins. It has been suggested that some orphan proteins may help plant viruses to infect and colonize its arthropod host/vector (Kuchibhatla et al., 2014; Solovyev and Morozov, 2017). Based on this same hypothesis, it has been speculated that kitaviruses have two sets of movement genes, and specifically cileviruses have the MP gene in conjunction with the p24 (putative virion membrane protein) and p61 (putative glycoprotein) proteins involved in virus movement within the mite host (Solovyev and Morozov, 2017). Based on the genomic organization of HYBV, this new kitavirid represents a distinct member within the family *Kitaviridae* that shares genomic similarities with members of both *Cilevirus* and *Higrevirus* genera.

Phylogenetic analyses of all conserved protein products support the placement of HYBV within the *Kitaviridae* family, specifically in an intermediate position between the cilevirus and higrevirus clades (Figure 4 and Supplementary Figure 4). The sequence identities of the three conserved kitavirid proteins, namely RdRp, p24 and MP, of HYBV with kitavirid homologs was found to be low to moderate (<45%). A similar scenario is observed for HGSV-2, the sole *Higrevirus* member and members of the genus *Blunervirus* (<38%). Conversely, the *Cilevirus* proteins have high identities (>54%) (Table 1 and Supplementary Figure 2). The relatively high protein identity levels among the *Cilevirus* protein homologs and the phylogenetic relatedness may suggest a more recent divergence within the genus, whereas blunerviruses diverged much earlier or might have undergone a more rapid evolution due to host or vector selective pressures. Considering the low protein identity level in the genus *Blunervirus*, it is plausible that in the future when more viruses belonging to the blunervirus clade are characterized, the genus may be divided into additional genera. The phylogenetic placement of HYBV, coupled with its distinctive genome organization, suggests this virus represents a distinct lineage intermediate of cileviruses and higreviruses. However, until more kitavirids are characterized which will allow greater resolution of the family’s taxonomy, it seems appropriate to consider HYBV a basal member of the genus *Cilevirus*, as it shares the most features with members of this taxon.

Multiple nucleotide sequence alignments of the 5′ and 3′ UTR revealed that HYBV possesses long conserved 5′ and 3′ UTRs rich in AT among its genomic segments (Supplementary Table 2), a feature shared by other cileviruses, most notably CiLV-C. Both 5′ and 3′ termini of HYBV RNA 1 and 2 were also highly conserved, with the latter being longer and sharing a higher nucleotide identity. Previously, it was found that the blunervirus BNRBV contains AT-rich UTRs and conserved termini (Quito-Avila et al., 2013). Also, the formation of stem-loop secondary structures in the 3′ UTR of the four RNAs was predicted and may be associated to the regulation of virus genome replication as well as protein synthesis (Quito-Avila et al., 2013). Interestingly, most of the cileviruses, HYBV, HGSV-2 and BNRBV have a G/C, and C as their first and last nucleotide, respectively (Supplementary Table 2). While sequencing clones for the 5′ RACE experiments for HYBV RNA 1 and 2, some indicated G (rather than the more common C) as the first nucleotide (data not shown). Considering the multiple plant hosts, limited *Brevipalpus* species associated to kitavirids, and conserved UTRs in some kitavirids, it is plausible that these regions may play a role in regulation of different virus infectious cycle processes within their hosts.

Flat mites are polyphagous and the presence of several *Brevipalpus* species on the same host plant has been reported previously (Salinas-Vargas et al., 2016). DNA barcoding using dRT-PCR (Druciarek et al., 2019) targeting the 28S rRNA and COI indicated mites tentatively identified as *B. yothersi* and an unidentified *Brevipalpus* sp. were present on the HYBV-infected *H. rosa-sinensis* plant. For this latter specimen, the COI sequence suggested its identity to be *B. obovatus*, however, the 28S rRNA sequence provided no clear identity, with resemblance to sequences attributed to *B. azores* and *B. feresi*. *B. yothersi*, *B. azores* and *B. feresi* are three of the seven recently created *Brevipalpus* species that were derived from the *B. phoenicis* species complex (Beard et al., 2015). Therefore, the identity of this *Brevipalpus* specimen needs further identification based on morphological keys using scanning electron microscopy (Beard et al., 2013). *B. yothersi* has been reported as the main vector of CiLV-C and CiLV-C2 (Roy et al., 2013; García-Escamilla et al., 2018; Ferreira et al., 2020). In this study we demonstrated the ingestion and potential acquisition of HYBV by *B. yothersi* using dRT-PCR assays targeting both RNA 1 and 2 of the virus. Considering the low number of individual *Brevipalpus* used in this study, the non-systemic nature of infection, and the ability of several *Brevipalpus* species to vector CiLV-C (Nunes et al., 2018), it is plausible that the other *Brevipalpus* sp. that could not be identified by DNA barcoding in this study may play a role in the transmission of HYBV. Additional transmission experiments are required to validate the transmission of HYBV by *B. yothersi* and other *Brevipalpus* species, and include confirmation of the mite species.

High-throughput sequencing data indicated that HCRSV and HLFPV were co-infecting the *H. rosa-sinensis* plant harboring HYBV. HCRSV infection has been associated with distinct mild ringspot symptoms and HLFPV with a latent infection (Kamenova and Adkins, 2004; Zhou et al., 2006). Neither of these are consistent with the observed symptoms of yellow chlorotic blotches with a dark green perimeter that are typical of a BTV infection (Figure 1 and Supplementary Figure 6), making HYBV the most likely causal agent of the observed symptoms. However, the presence of HCRSV and HLFPV, as well as the presence of CiLV-C2, as co-infections with HYBV may impact host symptoms and other aspects of this pathosystem. The development of an infectious clone of HYBV would greatly help elucidate its role as a causal agent of the observed disease. Until such a clone is available, the conventional and quantitative RT-PCR assays developed in this study for the detection of HYBV will help to further determine any relationship between HYBV infection, co-infection with other pathogens, and symptom expression. Although the presence of non-viral pathogens or physiological disorders cannot be excluded, the observed symptoms were absent from other hibiscus plants in the immediate area that would presumably be exposed to the same pathogen inoculum and growing conditions as the symptomatic plant. Additional symptomatic plants were observed up to 1.6 km away, some of which tested negative for HYBV, CiLV-C2, and HGSV-2 (Supplementary Figure 6). This suggests additional related viruses may be present in Hawaii’s hibiscus, adding to the complexity of this pathosystem.

## Data Availability Statement

The datasets presented in this study can be found in online repositories. The names of the repository/repositories and accession number(s) can be found below: https://www.ncbi.nlm.nih.gov/genbank/, MT472637 and MT472638.

## Author Contributions

AO-V and MM worked on the investigation, methodology, formal analyses, and supervised the study. AO-V and MM conceptualized and wrote the original draft. AO-V, JH, and MM wrote, reviewed, and edited the manuscript. MM was responsible for funding acquisition. MM and JH were responsible for the resources. All authors contributed to the article and approved the submitted version.

## Conflict of Interest

The authors declare that the research was conducted in the absence of any commercial or financial relationships that could be construed as a potential conflict of interest.

## Footnotes

1 ftp://ftp.ncbi.nih.gov/genomes/Viruses/all.fna.tar.gz

2 www.ncbi.nlm.nih.gov/orffinder

3 www.ncbi.nlm.nih.gov/Structure/cdd/wrpsb.cgi

4 www.ebi.ac.uk/Tools/hmmer/search/hmmscan

5 www.cbs.dtu.dk/services/SignalP-5.0/

6 http://tcoffee.crg.cat/apps/tcoffee/do:expresso

7 www.ebi.ac.uk/Tools/psa/lalign

8 https://ngphylogeny.fr

## Supplementary Material

Supplementary Figure 1 Agarose gel electrophoresis of dsRNAs extracted from hibiscus yellow blotch virus (HYBV)-infected *Hibiscus rosa-sinensis* leaves (right lane). Blue arrows at ∼8 Kbp and ∼4.5 Kbp indicate the observed dsRNAs that likely represent RNA 1 and RNA 2 of HYBV, respectively. The black arrow at ∼6 Kbp likely represents the genome of hibiscus latent Fort Pierce virus. Numbers on left give size in kilobase pairs (Kbp) for select fragments of the Thermo Fisher 1 kb Plus ladder (left lane).

Supplementary Figure 2 Pairwise protein sequence similarity matrix of the replication-associated polyprotein, p24 and putative movement protein of hibiscus yellow blotch virus (HYBV) with their homologs of *Cilevirus*, *Higrevirus*, and *Blunervirus* members using sequence demarcation tool (SDT) 1.2. Cilevirus species and isolates include: citrus leprosis virus C (CiLV-C) and CiLV-C2 (Citrus isolate from Colombia: Citrus-Col. Hibiscus isolate from Hawaii: HibiscusHI), and the proposed cilevirus, passion fruit green spot virus (PfGSV). Higrevirus member: hibiscus green spot virus 2 (HGSV2). Blunerviruses include: blueberry necrotic ring blotch virus (BNRBV) and tea plant necrotic ring blotch virus (TPNRBV), and the proposed blunervirus, tomato fruit blotch virus (TFBV).

Supplementary Figure 3 Structural alignment results of hibiscus yellow blotch virus (HYBV) p10 and hibiscus green spot virus 2 (HGSV-2) p10 using the Expresso algorithm implemented in T-Coffee. **(A)** Presence of two transmembrane helices in both HYBV-p10 and HGSV-2-p10 proteins and visualization of the structural alignment. **(B)** Expresso T-Coffee matrix result showing a significant score for the alignment (>50) and at least three structurally conserved motifs are observed in the alignment (Highlighted in light red). **(C)** Transitive consistency score of the protein alignment showing congruent structurally conserved positions revealed by Expresso-T-Coffee.

Supplementary Figure 4 Phylogenetic placement of hibiscus yellow blotch virus (HYBV) with other viruses possessing conserved proteins. **(A)** MP, putative movement protein (p32 of HYBV); **(B)** p61, putative glycoprotein (p56 of HYBV) and p29, putative coat protein (p33 of HYBV); and **(C)** p24, putative virion membrane protein (p25 of HYBV). All phylogenies were inferred by a multiple protein alignment using CLUSTAL and the maximum likelihood algorithm implemented in MEGA 7.0.25. Bootstrap values generated by maximum likelihood are shown above the branches after 1000 repetitions. Posterior probabilities that were calculated using Bayesian inference with three Markov Chain Monte Carlo runs of 10,000,000 generations and implemented in BEAST 2 are shown under the branches. Missing values indicate values below 50 (bootstrap) or 0.7 (posterior probability).

Supplementary Figure 5 Agarose gel electrophoresis of 28S rRNA, hibiscus yellow blotch (HYBV) RNA 1 and RNA 2 amplicons using direct RT-PCR (dRT-PCR) assays described by Druciarek et al. (2019). dRT-PCR assays were performed on four individual *Brevipalpus* mites (B1–B4) collected from a HYBV-infected *Hibiscus rosa-sinensis* plant. Upper panel: 28S rRNA and HYBV-RNA 1 amplicons were amplified using primer sets D1D2w2/28Sr0990 and HYBV-RdRp-F/R with expected amplicon sizes of ∼700 and 223 bp, respectively. Bottom panel: HYBV-RNA 1 and RNA2 amplicons were amplified using primer sets HYBV-p10-F/R and HYBV-p33-F/R with expected amplicons of 210 and 438 bp, respectively. A non-template control (N) was included in all the assays. Sanger sequencing of the 28S rRNA amplicon later suggested mites B1 and B2 were *B. yothersi*, while the identity of mites B3 and B4 was ambiguous. DNA ladder is 100 bp (Thermo Fisher), and sizes of select fragments are provided in base pairs (bp).

Supplementary Figure 6 Leaves collected from symptomatic *Hibiscus* spp. plants growing with 1.6 km of the hibiscus yellow blotch virus (HYBV)-infected *H. rosa-sinensis* plant. Samples A, B, and D tested positive for HYBV using RT-PCR, whereas Samples C, E, and F tested negative. Sample A also tested positive for citrus leprosis virus C2 by RT-PCR. None of the samples **(A–F)** tested positive for hibiscus green spot virus 2 by RT-PCR.

Supplementary Figure 7 Detection of hibiscus yellow blotch virus (HYBV), citrus leprosis virus C2 (CiLV-C2), and hibiscus green spot virus 2 (HGSV2) in *Hibiscus* spp. samples by RT-qPCR and RT-PCR assays. Six samples (A–F) of *Hibiscus* spp. (Supplementary Figure 3) collected within 1.6 km of the HYBV-infected *H. rosa-sinensis* underwent both assays using HYBV-RdRp-F/R (main text), CiLV-C2-RdRp-F/R and HGSV2-RdRp-F/R (Supplementary Table 1) for virus detection. Samples A, B, and D tested positive for HYBV in both one-step RT-qPCR **(A)** and two-step RT-PCR **(B)** assays. Due to similar amplification curve of samples A (dark red), B (blue), and D (light green), individual amplification curves are provided for each sample and compared with a positive control (red). Sample A was the only CiLV-C2-positive sample in both one-step RT-qPCR **(A)** and two-step RT-PCR **(B)** assays. No samples tested positive for HGSV2. Expected size of amplicons for HYBV, CiLV-C2, and HGSV2 are 223 bp, 386 bp, and 227 bp, respectively. NTC and (N) represent non-template controls for **(A)** and **(B)**, respectively. The DNA ladder in panel **(B)** is the 100 bp Ladder from Thermo Fisher, and fragment sizes are provided at left in base pairs (bp).

Supplementary Table 1 Primers used in this study for 5′ and 3′ RACE, genomic sequence validation, and detection of hibiscus yellow blotch virus (HYBV). Primers used for the detection of citrus leprosis virus C2 (CiLV-C2) and hibiscus green spot virus 2 (HGSV-2) in one-step RT-qPCR assays are also provided.

Supplementary Table 2 Kitavirus intra-species nucleotide composition analysis of the 5′ and 3′ untranslated regions (UTR), and 5′ and 3′ conserved termini among the genomic segments of each virus species.
