# Supplementary figures and images for: A Virus Infecting Hibiscus rosa-sinensis Represents an Evolutionary Link Between Cileviruses and Higreviruses
Source: Front Microbiol. 2021 May 3;12:660237. doi: 10.3389/fmicb.2021.660237 (PMC8126721; doi:10.3389/fmicb.2021.660237)

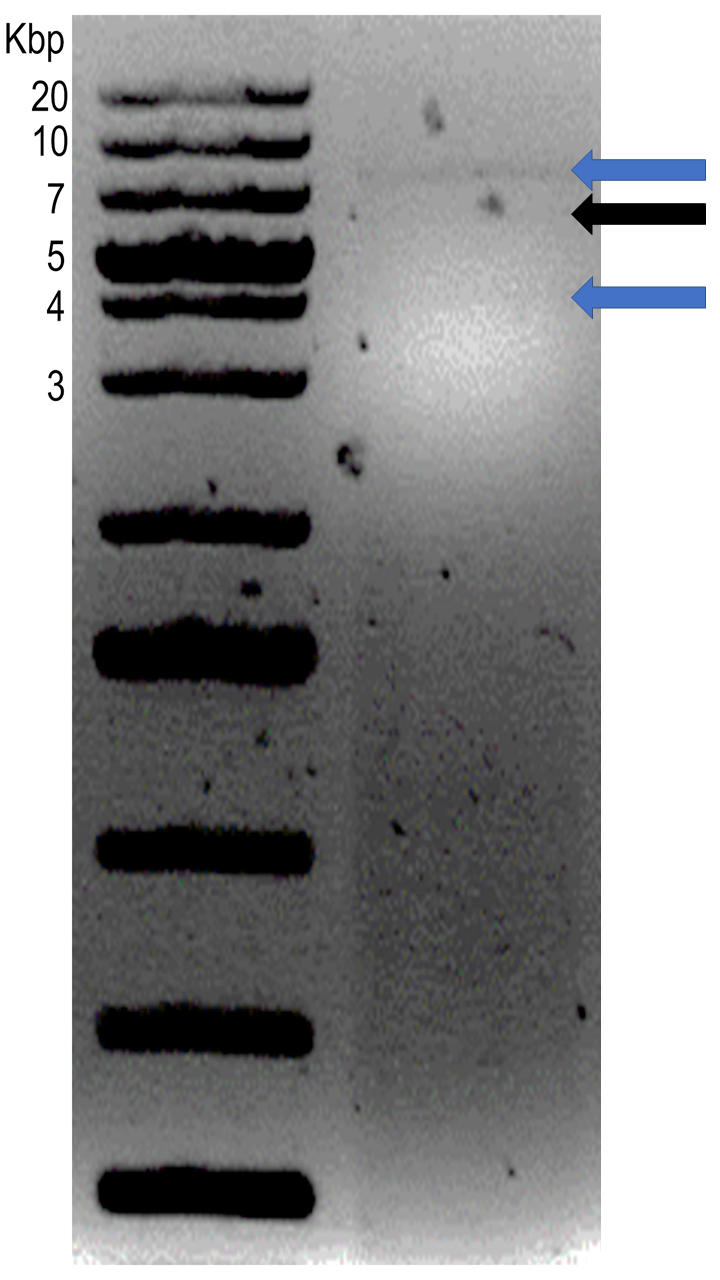

Supplement: Supplementary Figure 1 — Agarose gel electrophoresis of dsRNAs extracted from hibiscus yellow blotch virus (HYBV)-infected Hibiscus rosa-sinensis leaves (right lane). Blue arrows at ∼8 Kbp and ∼4.5 Kbp indicate the observed dsRNAs that likely represent RNA 1 and RNA 2 of HYBV, respectively. The black arrow at ∼6 Kbp likely represents the genome of hibiscus latent Fort Pierce virus. Numbers on left give size in kilobase pairs (Kbp) for select fragments of the Thermo Fisher 1 kb Plus ladder (left lane). [file Image_1.TIF]

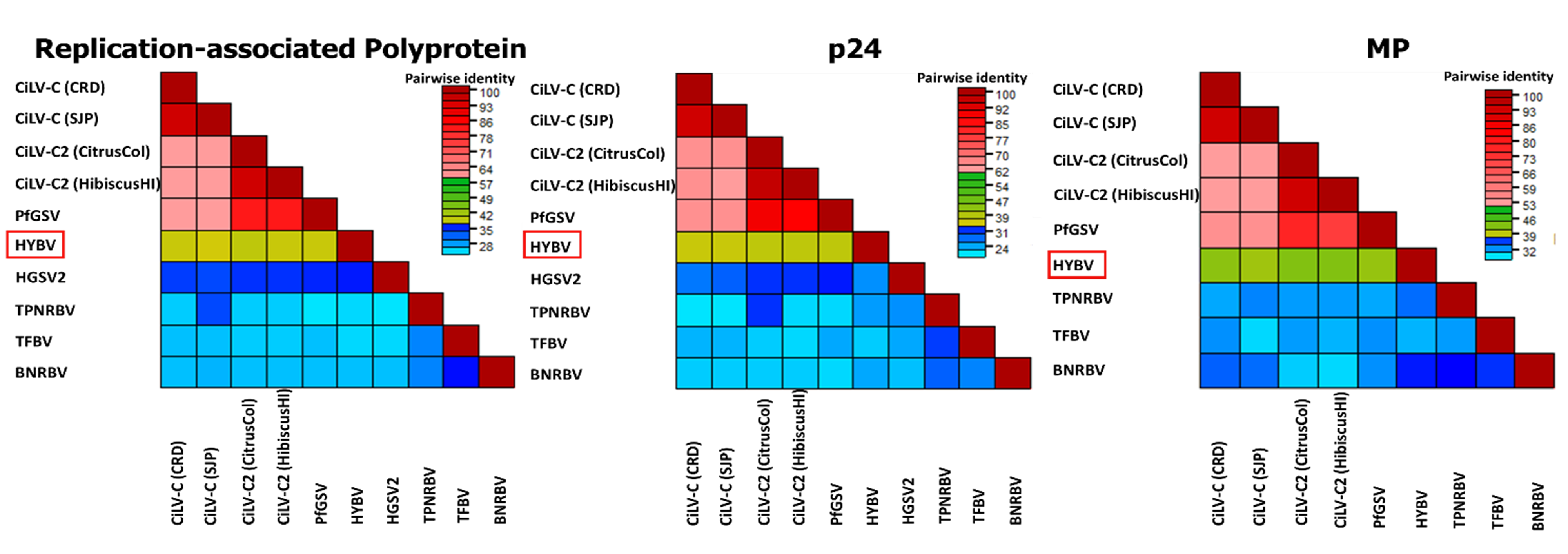

Supplement: Supplementary Figure 2 — Pairwise protein sequence similarity matrix of the replication-associated polyprotein, p24 and putative movement protein of hibiscus yellow blotch virus (HYBV) with their homologs of Cilevirus, Higrevirus, and Blunervirus members using sequence demarcation tool (SDT) 1.2. Cilevirus species and isolates include: citrus leprosis virus C (CiLV-C) and CiLV-C2 (Citrus isolate from Colombia: Citrus-Col. Hibiscus isolate from Hawaii: HibiscusHI), and the proposed cilevirus, passion fruit green spot virus (PfGSV). Higrevirus member: hibiscus green spot virus 2 (HGSV2). Blunerviruses include: blueberry necrotic ring blotch virus (BNRBV) and tea plant necrotic ring blotch virus (TPNRBV), and the proposed blunervirus, tomato fruit blotch virus (TFBV). [file Image_2.TIF]

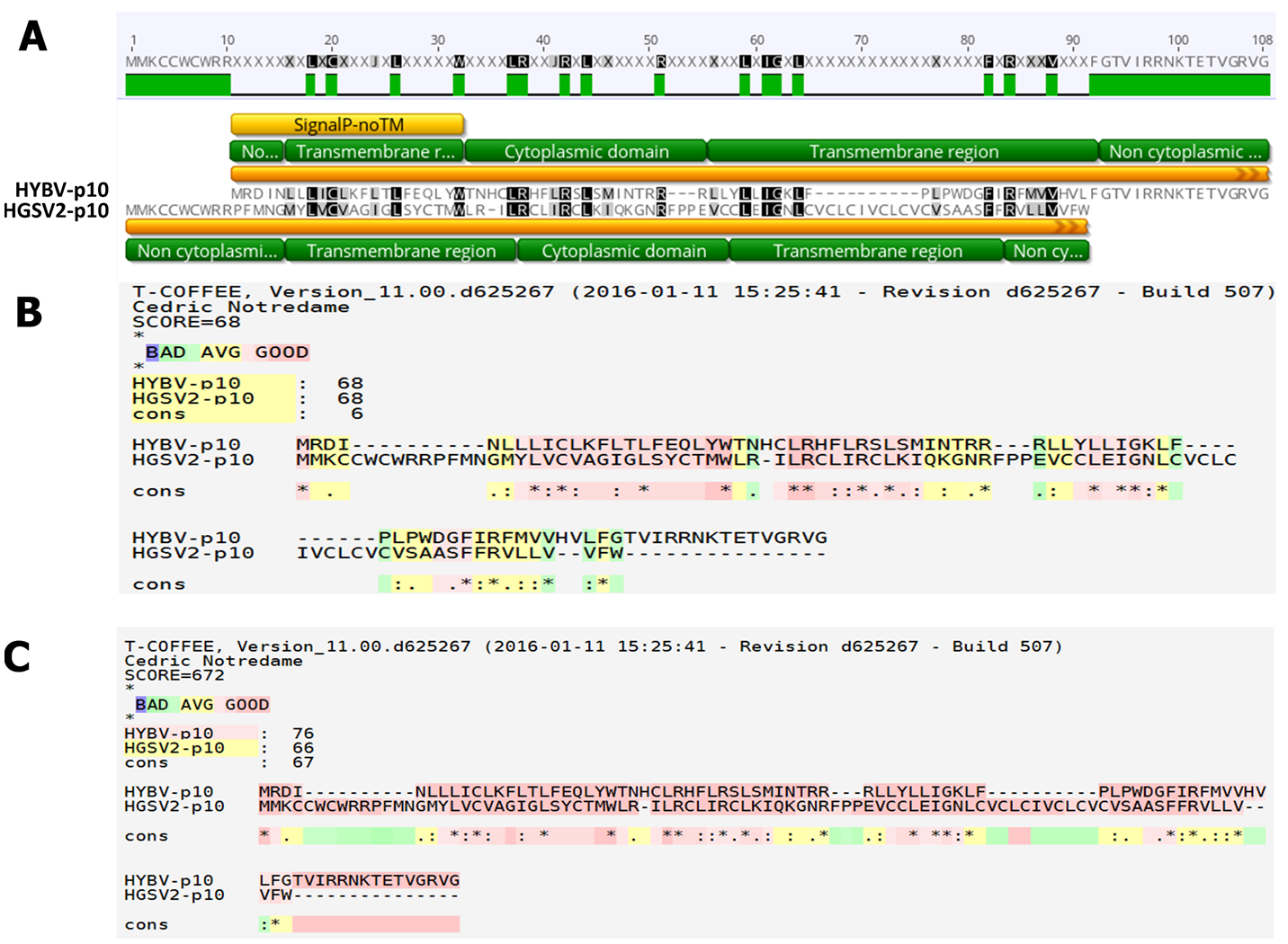

Supplement: Supplementary Figure 3 — Structural alignment results of hibiscus yellow blotch virus (HYBV) p10 and hibiscus green spot virus 2 (HGSV-2) p10 using the Expresso algorithm implemented in T-Coffee. (A) Presence of two transmembrane helices in both HYBV-p10 and HGSV-2-p10 proteins and visualization of the structural alignment. (B) Expresso T-Coffee matrix result showing a significant score for the alignment (>50) and at least three structurally conserved motifs are observed in the alignment (Highlighted in light red). (C) Transitive consistency score of the protein alignment showing congruent structurally conserved positions revealed by Expresso-T-Coffee. [file Image_3.TIF]

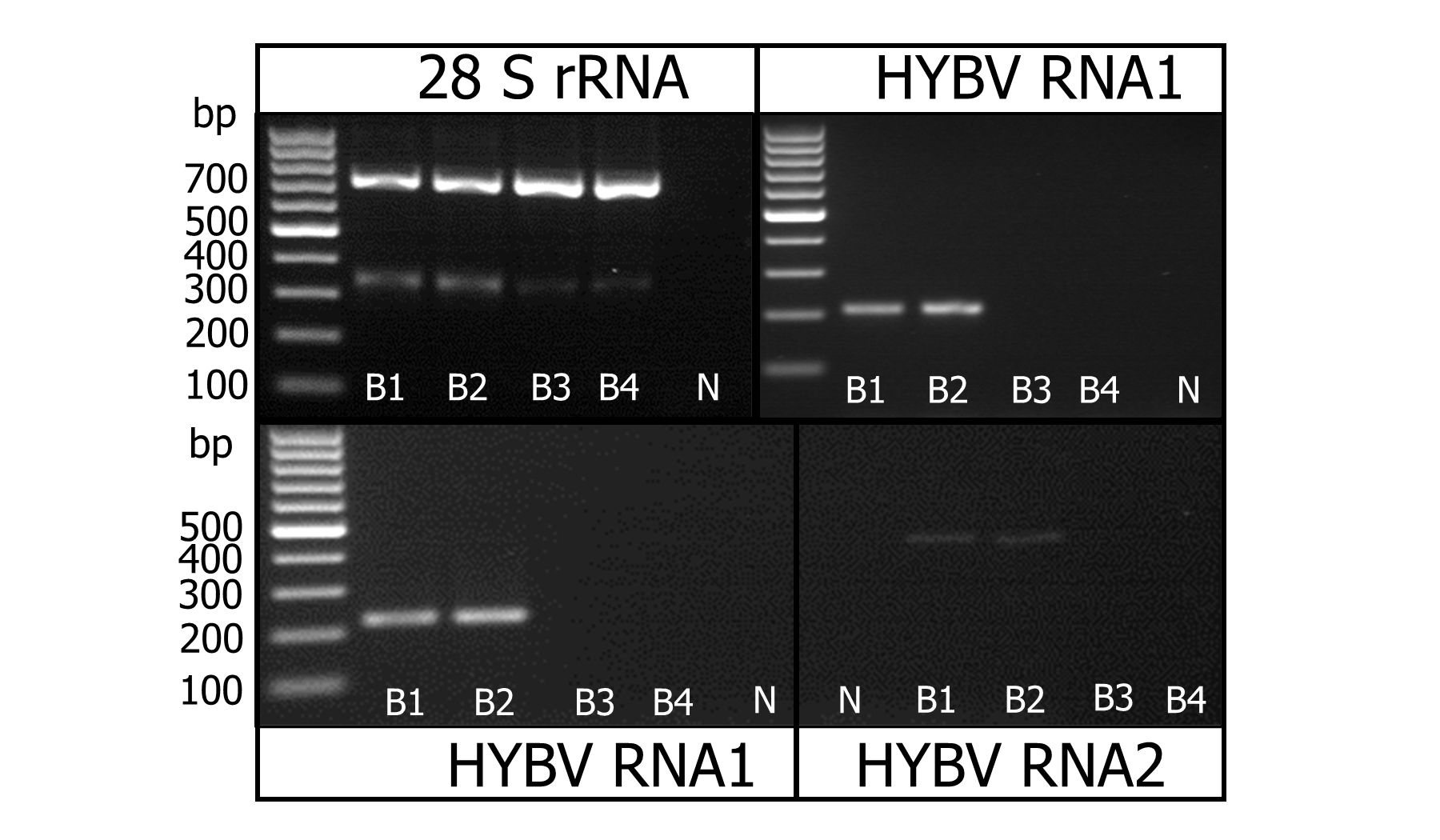

Supplement: Supplementary Figure 5 — Agarose gel electrophoresis of 28S rRNA, hibiscus yellow blotch (HYBV) RNA 1 and RNA 2 amplicons using direct RT-PCR (dRT-PCR) assays described by Druciarek et al. (2019). dRT-PCR assays were performed on four individual Brevipalpus mites (B1–B4) collected from a HYBV-infected Hibiscus rosa-sinensis plant. Upper panel: 28S rRNA and HYBV-RNA 1 amplicons were amplified using primer sets D1D2w2/28Sr0990 and HYBV-RdRp-F/R with expected amplicon sizes of ∼700 and 223 bp, respectively. Bottom panel: HYBV-RNA 1 and RNA2 amplicons were amplified using primer sets HYBV-p10-F/R and HYBV-p33-F/R with expected amplicons of 210 and 438 bp, respectively. A non-template control (N) was included in all the assays. Sanger sequencing of the 28S rRNA amplicon later suggested mites B1 and B2 were B. yothersi, while the identity of mites B3 and B4 was ambiguous. DNA ladder is 100 bp (Thermo Fisher), and sizes of select fragments are provided in base pairs (bp). [file Image_5.TIF]

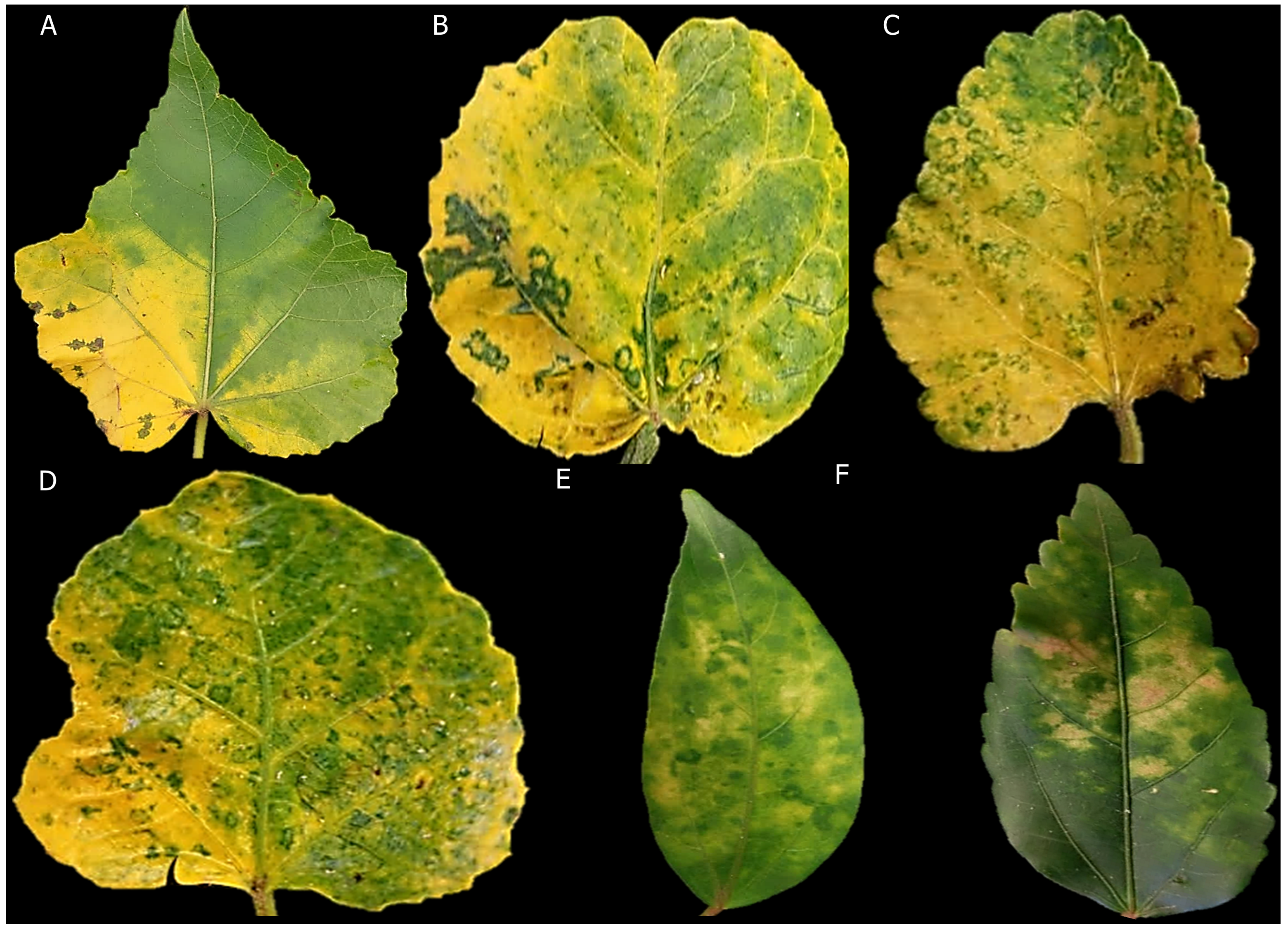

Supplement: Supplementary Figure 6 — Leaves collected from symptomatic Hibiscus spp. plants growing with 1.6 km of the hibiscus yellow blotch virus (HYBV)-infected H. rosa-sinensis plant. Samples A, B, and D tested positive for HYBV using RT-PCR, whereas Samples C, E, and F tested negative. Sample A also tested positive for citrus leprosis virus C2 by RT-PCR. None of the samples (A–F) tested positive for hibiscus green spot virus 2 by RT-PCR. [file Image_6.TIF]

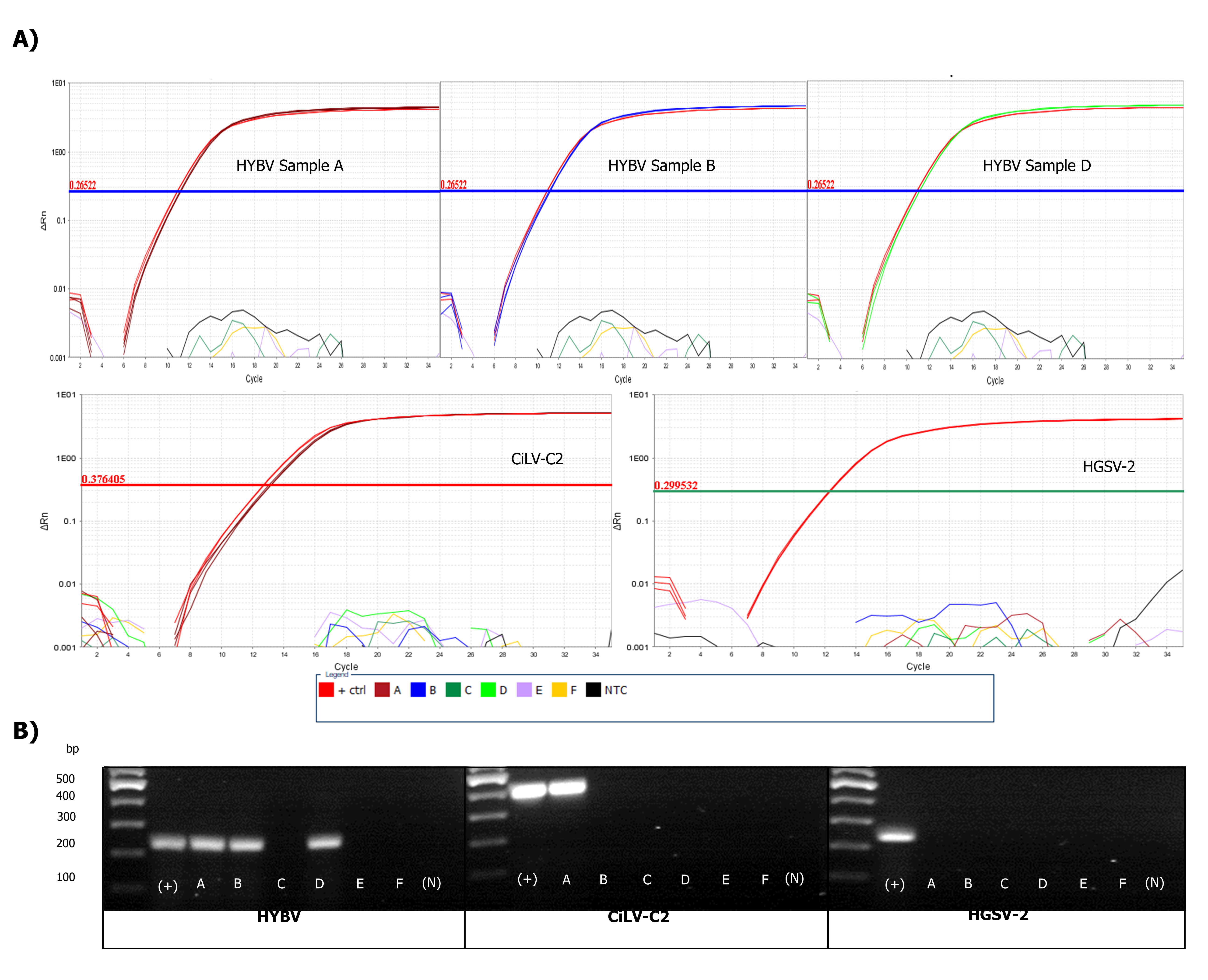

Supplement: Supplementary Figure 7 — Detection of hibiscus yellow blotch virus (HYBV), citrus leprosis virus C2 (CiLV-C2), and hibiscus green spot virus 2 (HGSV2) in Hibiscus spp. samples by RT-qPCR and RT-PCR assays. Six samples (A–F) of Hibiscus spp. (Supplementary Figure 3) collected within 1.6 km of the HYBV-infected H. rosa-sinensis underwent both assays using HYBV-RdRp-F/R (main text), CiLV-C2-RdRp-F/R and HGSV2-RdRp-F/R (Supplementary Table 1) for virus detection. Samples A, B, and D tested positive for HYBV in both one-step RT-qPCR (A) and two-step RT-PCR (B) assays. Due to similar amplification curve of samples A (dark red), B (blue), and D (light green), individual amplification curves are provided for each sample and compared with a positive control (red). Sample A was the only CiLV-C2-positive sample in both one-step RT-qPCR (A) and two-step RT-PCR (B) assays. No samples tested positive for HGSV2. Expected size of amplicons for HYBV, CiLV-C2, and HGSV2 are 223 bp, 386 bp, and 227 bp, respectively. NTC and (N) represent non-template controls for (A) and (B), respectively. The DNA ladder in panel (B) is the 100 bp Ladder from Thermo Fisher, and fragment sizes are provided at left in base pairs (bp). [file Image_7.TIF]
