# Supplementary material for: A Virus Infecting Hibiscus rosa-sinensis Represents an Evolutionary Link Between Cileviruses and Higreviruses
Source: Front Microbiol. 2021 May 3;12:660237. doi: 10.3389/fmicb.2021.660237 (PMC8126721; doi:10.3389/fmicb.2021.660237)
Supplement: Supplementary Table 1 — Primers used in this study for 5′ and 3′ RACE, genomic sequence validation, and detection of hibiscus yellow blotch virus (HYBV). Primers used for the detection of citrus leprosis virus C2 (CiLV-C2) and hibiscus green spot virus 2 (HGSV-2) in one-step RT-qPCR assays are also provided. [file Table_1.docx]

# Table S1. Primers used in this study for 5’ and 3’ RACE, genomic sequence validation, and detection of hibiscus yellow blotch virus (HYBV). Primers used for the detection of citrus leprosis virus C2 (CiLV-C2) and hibiscus green spot virus 2 (HGSV-2) in one-step RT-qPCR assays are also provided.

|  |  |  |  |
| --- | --- | --- | --- |
| **Name** | **Sequence (5' - 3')** | **Target** | **Purpose** |
| 744 | GACCACGCGACGTGTCGAVTTTTTTTTTTTTTTT | poly-A tail | 5' and 3' RACE |
| 743 | TTACTAATATCCCCCCCCCCCC | poly dC tail | 5' RACE |
| 880 | CGCTTGCATCTGGAGGAGTT | RNA 1 | 5' RACE |
| 881 | GGTTGTGTGTCTCCTGGCA | RNA 1 | Genome validation |
| 882 | GCTCTGGCCGATATCATAGAATC | RNA 1 | Genome validation |
| 883 | GTCTCAAGTCTGTCACGTTACC | RNA 1 | Genome validation |
| 884 | TCGCAGTCTTCCACGAACTT | RNA 1 | Genome validation |
| 885 | ATACTCTGACTGGTCTAAACGAGATG | RNA 1 | Genome validation |
| 886 | CAGCAATCGATCTATAGAAACCCATAC | RNA 1 | Genome validation |
| 887 | GAGGCTTTTGAGGAGTTTGT | RNA 1 | Genome validation |
| 888 | CGCTTAAAGACGACAGTATCAGC | RNA 1 | Genome validation |
| 889 | TCTTTGGTTACTGGTATCGG | RNA 1 | Genome validation |
| 890 | TGCTGTATTGACCTCTCGGA | RNA 1 | Genome validation |
| 891 | GGAGCACCCTACAGATGTTG | RNA 1 | Genome validation |
| 892 | CCTTCAGGTAAATACGGAGC | RNA 1 | Genome validation |
| 893 | TGAGTTGCCACCCCTAGCTA | RNA 1 | Genome validation |
| 894 | CACTCCTGTATAACTCGTTCCGG | RNA 1 | Genome validation |
| 895 | AAACTGGTACCCTGATCTGG | RNA 1 | Genome validation |
| 896 | TGAGCTTCATACCATAGATCGATCAG | RNA 1 | Genome validation |
| 897 | CTGGTAAGCGTTCTCTCGAG | RNA 1 | Genome validation |
| 898 | TACCCAGTACGCCACACCA | RNA 1 | Genome validation |
| 899 | TGTGACCGACCCTTGGAGTA | RNA1 | 3' RACE |
| 900 | AGAACAACCTGAACACGAGA | RNA 2 | 5' RACE |
| 901 | TTCCGGGTACACGTATGGC | RNA 2 | Genome validation |
| 902 | AAACAATGGGTGCCGTGACA | RNA 2 | Genome validation |
| 903 | TGTCCCAATTGTGTATAGATGAGGT | RNA 2 | Genome validation |
| 904 | ACCTAGGTCCGTCAACAGATG | RNA 2 | Genome validation |
| 905 | CGCTTGCTGACTCCGCTTT | RNA 2 | Genome validation |
| 906 | TGGTCTTTACGGATTTAGCATTGG | RNA 2 | Genome validation |
| 909 | CTATTACTCCTACCGCAGAG | RNA 2 | Genome validation |
| HYBV-p33-R | GCCGAACCAGGGAACATGAT | RNA 2 | Genome validation and RT-PCR assays |
| HYBV-p33-F | ACCGGTGGCTAATTCTTCTG | RNA 2 | 3' RACE and RT-PCR assays |
| HYBV-p10-F | ACACTATTCGAGCAGTTGTATTGGA | RNA 1 | RT-PCR  assays |
| HYBV-p10-R | CACCCTCCCAACCGTTTCA | RNA 1 |  |
| HYBV-RdRp-F | TGTATGCGTCCTGTGCTGTC | RNA 1 | Detection of HYBV |
| HYBV-RdRp-R | ATTCTTTGAGCTCGGCAGGT | RNA1 1 |  |
| Cile-p15F | CCCTTWCAYGADWTATCVTGTRAWTG | Cilevirus-p15 | p15 amplification |
| CiLV-C2-RdRp-F | ACAAGATGGCGGACGAACTG | CiLV-C2 RNA 1 (RdRp) | Detection of CiLV-C2 on one-step RT-qPCR |
| CiLV-C2-RdRp-R | AGCCATGTCATCGGGATCCA |  |  |
| HGSV2-RdRp-F | GGTGCCCGTGTTGTCTCATT | HGSV2 RNA1 (RdRp) | Detection of HGSV2 on one-step RT-qPCR |
| HGSV2-RdRp-R | CGTCACACCACTCAGCAACA |  |  |
