# Supplementary material for: A Virus Infecting Hibiscus rosa-sinensis Represents an Evolutionary Link Between Cileviruses and Higreviruses
Source: Front Microbiol. 2021 May 3;12:660237. doi: 10.3389/fmicb.2021.660237 (PMC8126721; doi:10.3389/fmicb.2021.660237)
Supplement: Supplementary Table 2 — Kitavirus intra-species nucleotide composition analysis of the 5′ and 3′ untranslated regions (UTR), and 5′ and 3′ conserved termini among the genomic segments of each virus species. [file Table_2.docx]

**Table S2.** Kitavirus intra-species nucleotide composition analysis of the 5’ and 3’ untranslated regions (UTR), and 5’ and 3’ conserved termini among the genomic segments of each virus species.

|  |  |  |  |  |  |  |  |  |  |  |  |
| --- | --- | --- | --- | --- | --- | --- | --- | --- | --- | --- | --- |
| **Kitavirus species^1^** |  | **5' UTR** | | **Conserved 5' termini** | | | **3' UTR** | | **Conserved 3' termini** | | |
|  | **Genbank**  **Accession** | **AT%** | **Length (bp)** | **Length (bp)** | **Nucleotide identity (%)** | **Best consensus sequence in the last nucleotides^4^** | **AT%** | **Length (bp)** | **Length (bp)** | **Nucleotide identity (%)** | **Best consensus sequence in the last nucleotides^4^** |
| HYBV | MT472637-8 | 55.5 | 267-297 | 65 | 69.2 | CATAAACKAAGAGTAGACTYRCKGGTTG | 63.2 | 198-341 | 171 | 91.4 | AACMTCYTTTAGCTTTTGTKTTT-AAAAGCC |
| CiLV-C | DQ157465-6  DQ352194-5 | 69.9 | 65-108 | 108 | 61.1 | GATAAAWCT(A)RTCAA | 61.5 | 228-254 | 126 | 92.9 | TTWRWTTTTCTTTTCTTTTGTCT-TTA(T)GAC |
| CiLV-C2^2^ (Citrus) | JX000024-5 | 67.2 | 92-118 | 10 | 70.0 | ATARAAWSAA | 64.9 | 223-349 | 127 | 90.6 | (C)TTYCTTTTCTTTTT(TC)TTGT-CATTWTGAC |
| CiLV-C2^2^ (Hibiscus) | MG253804-5  KC626783-4 | 63.7 | 56-119 | 32 | 81.1 | CATAGAATC(AA)CRATASTTRCTAT | 64.5 | 222-346 | 126 | 97.9 | TTCTTTCTTTTCTTTTTTTGTCAT-TTTGAC |
| PfGSV | MK804171-2 | 66.5 | 108-149 | 19 | 57.9 | SATRRRR(A)TACAAAAYWTT | 68.3 | 240-307 | 119 | 86.6 | TYTTTMTTTTCTTTTCTTTTGTC-TMTA(T)GAC |
| *Cilevirus*^3^ |  | 67.5 | 56-149 | 6 | 77.2 | (S)ATRRR | 63.6 | 222-349 | 122 | 78.9 | TYW(V)(N)TYTTYTYTTYTYTTG-TCWHTW(T)GAC |
| HGSV2 | HQ852052-4 | 55.8 | 214-464 | 13 | 79.5 | CATAAAWTD(M)AAA | 63.9 | 114-196 | 78 | 69.4 | (T)TTSTHTTWTCYTTTCT(TC)(C)TWKTC(C)S(T)G(C) |
| BNRBV | JN651148-51  KC433316-9 | 60.2 | 48-412 | 7 | 91.8 | SACAAAT | 65.1 | 146-429 | 9 | 70.8 | WTTATWMYCG |
| TPNRBV | MG781152-5 | 54.6 | 96-383 | 12 | 70 | (TGGGGAA)TTR(C)S | 59.3 | 128-941 | 85 | 70.9 | TTADTAAGAT(AAC) |
| TFBV | MK517477-80 | 58.5 | 77-426 | 20 | 78.8 | RWTTAA(CA)(T)CWTMAATCMAC | 55.6 | 76-333 | 13 | 80 | CKTTY(ACTCAGCT) |

^1^ Virus names for the provided acronyms are detailed in the main text.

^2^ CiLV-C2 possesses notable differences in the RNA 2 for strains infecting citrus and hibiscus. Differences in the untranslated regions as well as conserved termini were identified.

^3^ *Cilevirus* was the only genus in the family *Kitaviridae* whose members possessed conserved termini. *Higrevirus* was not considered in the analysis as a genus because it currently contains only a single species. No clear conserved termini were found among *Blunervirus* members.

^4^ Nucleotides between parentheses are not present in either all genomic segments or all virus isolates. HYBV, cileviruses and HGSV2 presented long conserved 3’ termini and longer consensus sequences than 30 nucleotides were obtained, however, only 30 nucleotides in the conserved termini sequences are presented. D= A, G, or T; K=G or T; M= A or C; R=A or G; S=C or G; W=A or T; Y=C or T
